# Measuring Environmental Chemical Burden with Wristbands: Implications for Kidney Health Among Women in Rural Guatemala

**DOI:** 10.3390/toxics13090761

**Published:** 2025-09-08

**Authors:** Jaime Butler-Dawson, Grant Erlandson, Diana Jaramillo, Karely Villarreal Hernandez, Laura Calvimontes, Lyndsay Krisher, Miranda Dally, Stephen Brindley, Daniel Pilloni, Alex Cruz, Alison K. Bauer, Richard J. Johnson, Lee S. Newman, Joshua Schaeffer, John L. Adgate, Kim A. Anderson, Katherine A. James

**Affiliations:** 1 Department of Environmental and Occupational Health, Colorado School of Public Health, University of Colorado, Anschutz Campus, Aurora, CO 80045, USA; 2 Centers for Health Work and Environment, Colorado School of Public Health, University of Colorado, Anschutz Campus, Aurora, CO 80045, USA; 3 Department of Environmental and Radiological Health Sciences, Colorado State University, Fort Collins, CO 80523, USA; 4 Grupo Pantaleon, Guatemala City 01010, Guatemala; 5 Division of Renal Diseases and Hypertension, University of Colorado School of Medicine, Anschutz Medical Campus, Aurora, CO 80045, USA; 6 Department of Epidemiology, Colorado School of Public Health, University of Colorado, Anschutz Campus, Aurora, CO 80045, USA; 7 Division of Pulmonary Sciences and Critical Care Medicine, Department of Medicine, School of Medicine, University of Colorado, Anschutz Campus, Aurora, CO 80045, USA; 8 Department of Environmental and Molecular Toxicology, Oregon Status University, Corvallis, OR 97331, USA

**Keywords:** chemicals, workers, exposure, community, kidneys

## Abstract

Chronic kidney disease of unknown origin (CKDu) is a public health concern, particularly in agricultural communities, with multiple environmental exposures hypothesized as potential contributors. This study employed a targeted exposure assessment using personal silicone wristbands to characterize chemical exposures among women living and working in CKDu-affected regions of Guatemala. Participants wore wristbands for seven days, passively sampling air and dermal exposures. Overall, 45 wristbands were collected from 37 female participants (19 sugarcane workers and 18 community members). Of the 1530 chemicals measured using a single semi-quantitative method, 103 were detected, with an average of 27 chemicals per wristband (range: 16–40). Polycyclic aromatic hydrocarbon (PAH) levels were higher in community members’ wristbands, whereas workers exhibited higher exposure to pesticides (i.e., pendimethalin and fipronil). Workers had worse kidney function compared to community members, with almost half of the workers having an estimated glomerular filtration rate, eGFR, <90 mL/min/1.73 m^2^. Correlations were observed between kidney function markers and specific chemicals, with the strongest correlation between albumin-to-creatinine ratio and pyrene levels (ρ = 0.57, *p* < 0.01) among workers. Women in agricultural regions of Guatemala experience widespread exposure to diverse environmental chemicals, some of which may contribute to kidney function decline.

## 1. Introduction

Agricultural workers and residents living near agricultural lands are potentially exposed to a variety of different chemicals. Our knowledge of these frontline exposures has steadily advanced over the past few years and has highlighted a probable connection between environmental toxicants and nephrotoxicity, implicating chemicals as potential contributors to chronic kidney disease of unknown origin (CKDu). CKDu is increasingly recognized as a significant global health issue, disproportionately affecting agricultural communities, particularly in Latin America among high-risk worker populations.

The etiology of CKDu, by definition [1], is not associated with traditional risk factors such as diabetes, hypertension, aging, or glomerular disease [2,3,4,5,6]. Studies indicate that recurrent dehydration and heat stress may contribute to CKDu risk [7,8,9,10,11], while additional factors—including medication use, smoking, genetic predisposition, and environmental exposures—may also play roles in disease pathogenesis [12,13,14]. Key environmental exposures warranting further investigation include agrochemicals [15,16,17] and airborne particulate matter and its constituents (e.g., heavy metals, and polycyclic aromatic hydrocarbons [PAHs]) [18,19,20,21,22,23].

Understanding how environmental exposures affect female workers is critical. Although CKDu has been predominantly reported among male sugarcane workers in Central America, limited research has examined women, leaving their disease burden largely uncharacterized [12,24,25,26]. Women remain underrepresented in CKDu research despite evidence of adverse kidney outcomes [27] and similar exposure conditions. This study focuses on women’s occupational and non-occupational exposures to organic chemicals and their potential relationships with kidney health.

Women in the region are frequently exposed to airborne pollutants from indoor cooking, ambient air, and agricultural sources [28] and these pollutants may impact kidney health [29,30,31,32,33,34]. In Guatemala, many households cook using solid fuels, such as wood, often burned indoors on open fires, leading to elevated indoor air pollution [35]. Additionally, pre-harvest sugarcane burning releases airborne pollutants that may contribute to adverse health effects in both workers and surrounding communities [36,37,38,39]. Based on the burning and incomplete combustion, PAHs may also impact inflammation and kidney health [40]. One study conducted in El Salvador observed an association between CKDu prevalence and exposure to the organophosphate pesticide methyl parathion [30]. However, no comprehensive chemical exposure assessments have been conducted among women in Central America.

A broad and integrated exposure assessment is essential to understanding the role of chemicals in kidney health. Personal chemical exposure remains poorly characterized among populations at risk for CKDu, particularly among women. Silicone wristbands offer a noninvasive method for passive sampling of a wide range of environmental contaminants. These wristbands absorb volatile and semi-volatile chemicals from air, dust, and dermal contact, providing an integrated measure of inhalation and dermal exposure [41]. Prior studies have demonstrated their effectiveness in detecting PAHs, flame retardants, organophosphate esters, pesticides, and other environmental pollutants [42,43,44,45,46,47,48]. Furthermore, wristband measurements correlate with exposure biomarkers in human biospecimens (serum and urine) [45,49,50,51]. Although many wristband studies are exposure-focused, only a few have moved toward health-outcome analyses [52,53]. We are not aware of a study which examines the link between chemical exposure directly to kidney biomarkers.

This study investigated chemical exposures and their effects on kidney function among women working in sugarcane fields and those living in agricultural communities in Guatemala. Specifically, we deployed silicone wristbands to characterize chemical exposure profiles among two distinct female populations. Our objectives were to: (1) use a targeted approach to assess chemical exposures in communities affected by CKDu, (2) compare exposure patterns between workers and non-working community members, and (3) explore associations between frequently detected chemicals and kidney function markers. We hypothesized that exposure patterns would differ by occupation and that higher pesticide and PAH concentrations would be associated with worse kidney outcomes.

## 2. Material and Methods

### 2.1. Study Background and Population

This sub-study is part of a larger longitudinal investigation of environmental exposures and kidney health in Guatemalan agriculture communities during two consecutive six-month sugarcane harvest seasons [28]. Details of the study have been described previously by Butler-Dawson et al. (2024) [28]. The larger study included 54 participants, 22 female sugarcane workers (hereafter referred to as “workers”) and 32 female community members who were not employed in the sugarcane industry nor engaged in any work outside the home (hereafter referred to as “community members”). These two groups were selected to compare occupational exposures and non-occupational exposures. We recruited and obtained consent from all women field workers who were employed as seeders at one agribusiness in southwest Guatemala during the two study harvest seasons (2021–2022, 2022–2023). Workers participated either for one or two years depending on which harvest they worked. We recruited community members only during the second season (December 2022). They were recruited from communities adjacent to sugarcane fields, specifically in the Departments of Escuintla and Suchitepéquez, where sugarcane is the predominant crop. Community members were identified through a convenience sampling approach, relying on referrals from their spouses or neighbors, who were male sugarcane cutters employed by the agribusiness. All participants were aged 18 years or older and reported that they were not pregnant. Written informed consent was obtained from all participants. Ethical approval for the study was granted by the Colorado Multiple Institution Review Board (COMIRB) and ZUGUEME Comité Ética Independiente in Guatemala.

In this sub-study, we recruited 37 of the 54 participants (19 workers and 18 community members). The participants wore silicone wristbands to characterize organic chemical exposures from airborne sources, direct contact such as sweat on the skin, or through dermal excretion (e.g., caffeine) [43]. Eligibility criterion for participation in the sub-study was based on availability to receive a wristband seven days before the scheduled data collection days.

The participants in the wristband sub-study group (*n* = 37) did not differ significantly from participants who were not part of the sub-group (*n* = 17) in terms of age, body mass index (BMI), blood pressure, or kidney function markers. This sample size was sufficient to provide preliminary data on personal environmental chemical exposures and explore associations with kidney function markers, informing the design of a larger, targeted study.

### 2.2. Study Design

**Figure 1:**
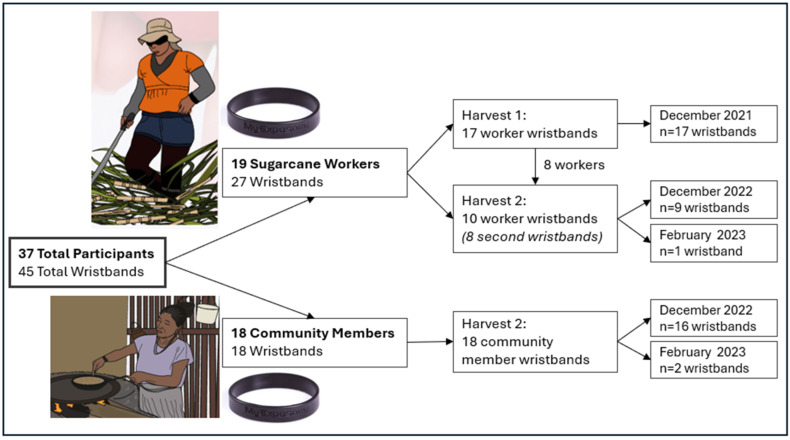
Study design across the two study harvests.

During the first harvest (December 2021), 19 workers wore pre-cleaned silicone wristbands, Figure 1. In the second harvest, ten workers participated, including eight returning workers and two new workers. The returning workers wore a new wristband during this period. Additionally, 18 community members each wore a single wristband during the second harvest. Wristbands were worn either in December 2022 or February 2023 during the second harvest period. Participants also provided urine and blood samples and completed a questionnaire.

#### 2.2.1. Wristband Deployment and Analysis

We used silicone wristbands provided and analyzed by MyExposome (MyExposome, Inc., Corvallis, OR, USA) [54]. Participants wore wristbands continuously on their dominant wrists for seven days. Prior to deployment, the wristbands were prepared using previously described methods [55]. Each wristband was enclosed in an individual airtight polytetrafluoroethylene (PTFE) bag provided by MyExposome and stored in these bags before and after use. Previous research has demonstrated that chemicals remain stable in wristbands for up to one month at +30 °C, minimizing the risk of degradation or diffusional losses in various settings [55,56].

Participants were instructed to wear wristbands throughout all their daily activities and while sleeping, avoiding direct application of lotion to the wristband. A seven-day deployment period was selected to capture a full weekly cycle of chemical exposures, including six workdays and one rest day for the workers. Community members were mostly not engaged in occupational activities during this period, minimizing day-to-day variability in exposures. Wristbands were worn over long-sleeved shirts for sugarcane workers during fieldwork and varied on non-work days; community members wore wristbands on the skin or over clothing depending on attire. On the seventh day, participants removed the wristbands, placed them back in their original PTFE bags, sealed the bags, and returned them to the research team. Five community participants did not retain their original packaging, so their wristbands were placed in Ziploc^®^ plastic bags (S. C. Johnson & Son, Racine, WI, USA). and sealed upon collection; these have been used in prior studies as an inert storage option for silicone wristbands [43]. No significant differences in chemical category frequencies or individual chemical detection rates were observed between wristbands returned in PTFE bags (*n* = 40) and those in alternative plastic bags (*n* = 5), as assessed by Fisher’s exact test.

Following collection in the field, wristbands were transported in a cooler and place in a freezer within one hour of collection. Wristbands were stored per manufacturer’s recommendation and then shipped overnight to MyExposome (MyExposome, Inc., Corvallis, OR, USA) for chemical analysis. Prior studies have demonstrated that chemical concentrations in silicone wristbands remain stable under a range of storage temperatures, including room temperature and freezing conditions, supporting their suitability for environmental exposure assessment in diverse field settings [43,55]. Wristbands were solvent-extracted following the method described by O’Connell et al. [57]. MyExposome performed an analytical screen for 1530 chemicals using an established gas chromatography–mass spectrometry (GC-MS) method, with details outlined in previous studies [55,57]. Wristband chemical concentrations are reported in units of ng/g wristband.

To facilitate comparisons with other studies, we used MyExposome’s chemical classification system. Categories included pesticides (e.g., permethrin, N,N-diethyl-m-toluamide), PAHs, volatile organic chemicals [VOCs], pharmacological chemicals (e.g., butylated hydroxyanisole), flame retardants (e.g., organophosphate esters), chemicals in commerce (e.g., phthalates, organophosphate esters), personal care products (e.g., fragrances, UV-blockers), and consumer products (e.g., caffeine, scents, food preservatives). Several chemicals were assigned to multiple categories, see full list of detected chemicals in Supplemental Table S1. Five chemicals, bis [2-ethylhexyl] phthalate, di-n-butyl phthalate, diethyl phthalate, eugenol, and triethyl phosphate, were classified as both chemicals of commerce and pesticides. However, for this analysis, they were categorized solely as chemicals of commerce, as they function only occasionally as inert ingredients in pesticide formulations [58].

The analyzed chemical set included 76 consumer product-related chemicals, 124 flame retardants, 185 industrial-related chemicals, 98 PAHs, 260 PCBs/dioxins/furans, 773 pesticides and 14 phthalates. The complete list of chemicals is available at https://www.myexposome.com/fullscreen (accessed on 17 May 2025).

#### 2.2.2. Urine and Blood Collection

Urine and blood samples were collected on the seventh day of participation. Creatinine levels were immediately analyzed using a point-of-care handheld analyzer (i-STAT^®^ Abbott Point of Care, Princeton, NJ, USA), following the manufacturer’s recommendations. Remaining biological samples were transported on ice in a cooler to an independent, licensed clinical laboratory (Herrera Llerandi laboratory, Guatemala City, Guatemala). Blood HbA1c was determined using ionic-exchange high-pressure liquid chromatography (Biorad, D-10). Serum cystatin C values were determined using turbidimetry-based immunoassay. Estimated glomerular filtration rates (eGFR) were calculated using the 2021 Chronic Kidney Disease Epidemiology Collaboration (CKD-EPI) equations [59] incorporating both serum creatinine and cystatin C (eGFR-creatinine-cystatin C), which has been shown to improve accuracy estimates of kidney function compared with using creatinine alone, especially across diverse populations [59]. We defined three categories of kidney function based on eGFR results: moderate kidney dysfunction (< 60 mL/min/1.7 m^2^), reduced kidney dysfunction (60–90 mL/min/1.7 m^2^), and normal (≥90 mL/min/1.7 m^2^). For comparability with prior studies, we also report eGFR-creatinine estimates.

Urine samples were analyzed for albumin and creatinine, and the urinary albumin-to-creatinine ratio (ACR) was calculated. Urinary creatinine was measured using the kinetic alkaline picrate method, and urine sodium concentrations were determined via an ion-selective method using an automatic biochemical analyzer (Roche Cobas Integra 400 Plus, Roche Diagnostics, Indianapolis, IN, USA). Urinary albumin was measured using florescence immunoassay (Boditech, I-Chroma).

#### 2.2.3. Questionnaires

Self-reported information on participant characteristics, including demographic data, work history, health history, medication use, household agricultural exposure, and lifestyle factors during the seven-day wristband deployment, was collected through interviewer-administered questionnaires. Participants were asked about activities such as travel outside their community, agrochemical or pesticide use, tobacco product use, household tobacco exposure, personal care product use, and wristband compliance. Given the potential relevance of personal care products to phthalate exposure, participants reported their use of body oil, lotion, and nail polish. Additionally, household cooking practices, including fuel type, stove location, and primary cook status, were documented.

### 2.3. Statistical Analysis

We calculated descriptive statistics of personal characteristics and health data stratified by group (i.e., workers vs. community members). To compare differences in chemical category detection frequencies and the average number of chemicals detected per category between groups, we used unadjusted linear or logistic mixed-effects regression models, which included participant-level random intercepts to account for repeated measures within individuals (*n* = 8). Model assumptions, including normality of residuals, were evaluated for all mixed-effects models, and no major deviations were observed.

Descriptive statistics were then calculated for the 103 chemicals detected in silicone wristbands. Analyses of individual chemicals were primarily observational due to the small sample size. Non-detects were excluded, and analyses were restricted to concentrations above detection limits. To reduce concerns with multiple testing, analyses initially focused on chemicals detected in at least 30% of wristbands in either group (Subset 1 Chemicals, *n* = 41). Preliminary analyses suggested that wristband concentrations were log-normally distributed; thus, concentrations were log-transformed for subsequent analyses. Differences in chemical presence/absence and log-transformed concentrations between groups were assessed using mixed-effects regression models with Subset 1 Chemicals.

For chemicals detected in at least 50% of wristbands, called Subset 2 Chemicals (*n* = 17 chemicals), relationships between log-transformed concentrations and potential exposure determinants and baseline characteristics were examined using the non-parametric Wilcoxon rank sum test, considering only the first wristband worn by each participant (*n* = 37). Predefined exposure-chemical relationships were evaluated with the Subset 2 Chemical, including: (1) self-reported use of nail polish and lotion/body oil in relation to triphenyl phosphate (TPP), di-n-butyl phthalate, diisobutyl phthalate, butyl benzyl phthalate, di-n-nonyl phthalate, galaxolide, tonalide, and diethyl phthalate concentrations [60,61,62]; (2) department, tobacco use, household tobacco use, and cooking-related practices in relation to PAH concentrations; and (3) self-reported medication use in relation to pharmacological concentrations. No pesticides were included in Subset 2 Chemicals, so self-reported agricultural practices and agrochemical use were not assessed.

Markers of kidney function were compared between groups using the non-parametric Wilcoxon rank sum test. Preliminary associations between chemicals and markers of kidney function (i.e., ACR, eGFR-creatinine, eGFR-creatinine-cystatin C) were explored using non-parametric Spearman correlation coefficients, stratified by group. This correlation analysis was limited to the 17 log-transformed chemicals detected in at least 50% of wristbands (Subset 2 Chemicals). No corrections for multiple comparisons were applied, as this pilot study was intended to be hypothesis-generating; analyses focused on chemicals with ≥50% detection and non-parametric correlations to limit the risk of spurious associations. All analyses were performed using SAS statistical software (version 9.4; SAS Institute Inc., Cary, NC, USA).

## 3. Results

Participant demographics and characteristics are summarized in Table 1. Age ranged from 19 to 55 years, with an average of 34 years (SD: 10). All workers were from the Department of Escuintla, while community members were from Escuintla (56%) and neighboring Suchitepéquez (44%). Nearly all community members were the primary cooks in their households, with 59% cooking in a separate enclosed room which is not part of their main house. Among workers, two-thirds were primary cooks, with most cooking either outside (39%) or inside the main house (44%).

### 3.1. Chemical Measurements

Of 1530 chemicals measured, 103 chemicals were detected in at least one of the 45 wristbands (Supplemental Table S1). Seventeen chemicals (16.5%) were detected in at least 50% of wristbands (Supplemental Table S2). The minimum number of chemicals found on any one wristband was 16 and the maximum was 40. On average, 27 chemicals (SD: 5.5) were detected per wristband, with similar detection numbers between workers (26 chemicals, range: 16–39) and community members (28 chemicals, range: 20–40). The most detected compounds (≥95%) included fragrance chemicals (benzyl salicylate, galaxolide, lilial, tonalide), plasticizers (diisobutyl phthalate, bis [2-ethylhexyl] phthalate, diethyl phthalate), pyrene, and triphenyl phosphate, a flame retardant and plasticizer.

Quantifiable concentrations were found for 30 out of 63 PAHs tested (48%), 18 out of 75 pesticides (24%), and 8 out of 42 flame retardants (19%). Additional detected chemicals included pharmacological (*n* = 3), chemicals in commerce (*n* = 35), consumer products (*n* = 13), and personal care products (*n* = 27).

### 3.2. Chemical Categories Between Groups

Each of the wristbands contained at least one chemical from three chemical classes: PAHs, personal care products, and chemicals in commerce (Table 2). Workers’ wristbands detected a higher number of pesticides than the wristbands worn by community members (3.08 vs. 2.11, *p* < 0.01). Conversely, community members’ wristbands detected a higher number of PAHs than the workers’ wristbands (10.44 vs. 6.48, *p* < 0.01).

### 3.3. Individual Chemical Detections and Concentrations Between Participant Groups

Detection frequencies and log-transformed concentrations were compared for 41 chemicals detected in at least 30% of either group’s wristbands (Subset 1 Chemicals, Table 3). Distributions are visually presented for PAHs (Supplemental Figure S1), pesticides and phthalates (Supplemental Figure S2), and personal care products, chemicals of commerce, TPP and caffeine concentrations (Supplemental Figure S3).

#### 3.3.1. PAH Exposure

Of the 30 detected PAHs, 16 were found in at least 30% of wristbands. Pyrene (100%), anthracene (84%), and benz(a)anthracene (60%) were the most commonly detected PAHs. Community members had higher detection frequencies for 13 of 16 PAHs, with significant differences for benz(a)anthracene (94% vs. 37%), 1-methylnaphthalene (56% vs. 19%), fluorene (72% vs. 33%), benzo(a)pyrene (61% vs. 26%), and fluoranthene (61% vs. 26%). Naphthalene was detected more frequently in workers (41% vs. 11%).

Median PAH concentrations were highest for phenanthrene, pyrene, and fluoranthene. Community members had significantly higher concentrations of pyrene (116.6 vs. 68.7 ng/g, *p* = 0.01) and cyclopenta(cd)pyrene (44.9 vs. 22.5 ng/g, *p* = 0.03). Naphthalene concentrations were slightly higher for the community members’ than the workers’ wristbands (13.4 vs. 5.7 ng/g, respectively) although detected more frequently among the workers’ wristbands as noted above.

#### 3.3.2. Pesticide Exposure

Of the 18 detected pesticides overall, five were detected in at least 30% of either group’s wristbands, which included pendimethalin, benzyl benzoate, permethrin, fipronil, and biphenyl. Pendimethalin was detected in 63% of the workers’ wristbands and fipronil at 30%, while none of the community members’ wristbands had these two chemicals. Community members’ wristbands had significantly higher detection frequencies of biphenyl (67% vs. 26%). Pesticide concentrations did not differ significantly between groups.

#### 3.3.3. Phthalates and Chemicals of Commerce Exposure

Diisobutyl phthalate (100%), di-n-nonyl phthalate (69%), and butyl benzyl phthalate (55%) were commonly detected. Community members had higher detection frequencies of di-n-butyl phthalate (100% vs. 78%) and butyl benzyl phthalate (72% vs. 44%). Diuron metabolite [3,4-dichlorophenyl isocyanate], a chemical intermediate, was found only in workers (63%).

#### 3.3.4. Other Chemical Exposures

β-ionone was more frequently detected in community members (83% vs. 56%). Musk ketone and citral A were more frequent in workers’ wristbands (37% vs. 11% and 33% vs. 6%). The flame retardant and plasticizer, TPP, was detected in 98% of wristbands, with significantly higher log-transformed concentrations in community members’ wristbands (297 ng/g vs. 144 ng/g, *p* < 0.01). Among the consumer products, workers’ wristbands had significantly higher caffeine detection frequencies (85% vs. 44%) and concentrations (1360 ng/g vs. 352 ng/g, *p* < 0.01) compared to community members’ wristbands.

### 3.4. Determinants of Chemical Concentrations

We examined whether certain personal and household characteristics are related to specific log-transformed chemicals detected in ≥ 50% of the wristbands, Subset 2 Chemicals (Supplemental Table S2). We only used first wristbands (*n* = 37) with this specific analysis and excluded the eight additional wristbands from workers during the second study harvest. Many of the participants (87%) reported using lotion or body oils at least once during the seven-day sampling period. Four workers (21%) and no community members reported using nail polish. Personal care product use was associated with higher concentrations of benzyl salicylate (*p* = 0.07) and galaxolide (*p* = 0.03). Women who reported mainly cooking outside or in an enclosed space other than their house had higher pyrene levels compared to women who cooked inside their house (*p* = 0.01). Wood fuel (75%) was more commonly used than liquid fuel (25%) when cooking outside and those who cooked inside their main home used liquid fuel (79%) compared to wood fuel (21%).

We examined differences between Departments only among the community members. We observed significantly higher levels of TPP among community members who live in Escuintla compared to Suchitepéquez (*p* < 0.001), while those in Suchitepéquez had higher lilial (*p* = 0.02) and galaxolide (*p* < 0.01) concentrations.

### 3.5. Correlations Between Individual Chemicals and Markers of Kidney Function

**Figure 2:**
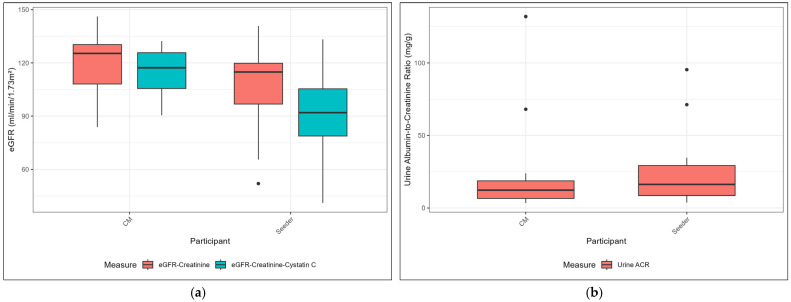
Box plots of kidney function markers by participant group: (**a**) estimated glomerular filtration rate (eGFR) for eGFR-creatinine and eGFR-creatinine-cystatin C. Values between 60–90 represent reduced kidney dysfunction. Values < 60 represent moderate kidney dysfunction. (**b**) Albumin-to-creatinine ratio (ACR) by group. Values > 30 represent elevated ACR levels.

Workers had worse kidney function compared to the community members, Table 4, Figure 2. Workers had significantly lower eGFR-creatinine-cystatin C (91 vs. 117 mL/min/1.73 m^2^). Almost half of the workers (47%) had an eGFR-creatinine-cystatin C < 90 mL/min/1.73 m^2^, with one worker at <60 mL/min/1.73 m^2^. While workers had higher average ACR values, the difference was not statistically significant.

We examined correlations between the Subset 2 Chemicals and markers of kidney function stratified by group (Table 5). Additionally, we examined two pesticides that were only in the Subset 1 Chemicals based on *a priori* hypotheses among the workers (i.e., pendimethalin, and benzyl benzoate). Among the community members, ACR had a slight positive correlation with diethyl phthalate (ρ = 0.44, *p* = 0.09). There were no observed correlations between eGFR-creatinine-cystatin C and the chemicals.

In workers, there was a weak negative correlation between eGFR-creatinine-cystatin C and butyl benzyl phthalate (ρ = −0.57, *p* = 0.05) and eGFR-creatinine was weakly negatively correlated with β-ionone (ρ = −0.40, *p* = 0.14). ACR was positively correlated with pyrene (ρ = 0.57, *p* = <0.01) and pendimethalin (ρ = 0.55, *p* = 0.03) and was weakly positively correlated with β-ionone (ρ = 0.43, *p* = 0.14) and benzyl salicylate (ρ = 0.34, *p* = 0.10).

## 4. Discussion

This study provides a comprehensive snapshot of chemical exposures among women in agricultural communities, revealing major differences in exposure profiles between workers and non-working community members. We identified 103 distinct chemicals; many not previously measured in this population and observed worse kidney function among agricultural workers. Despite the relatively small sample size, our findings highlight both occupational and household exposure pathways that may contribute to early kidney dysfunction. Given the small sample size, these findings are exploratory and intended to generate hypotheses for future studies examining associations between chemical exposures and kidney function. These results point to specific chemical classes and exposure sources that warrant further investigation in larger epidemiologic studies focused on chronic kidney disease risk.

While both groups had similar numbers of detected chemicals, wristbands worn by community members exhibited higher detection rates and higher concentrations of PAHs compared to workers’ wristbands. This finding is likely due to indoor cooking with biomass, whereas workers had greater detection of lighter PAHs like naphthalene, which is less associated with open-fire combustion and is more commonly linked to motor vehicle exhaust, potentially explaining its higher prevalence in workers, who are frequently exposed to sugarcane transport truck emissions during harvest [63]. Additional sources of naphthalene included moth preventatives, fumigants, and pesticides [63,64]. We collected survey information on cooking location, daily cooking time, fuel type, and stove type to capture household sources of PAH exposure; however, spatial data on proximity to sugarcane burning were not available, limiting our ability to distinguish between household and surrounding agricultural contributions.

Pyrene and anthracene were moderately correlated with higher ACR, consistent with their known nephrotoxic effects through oxidative stress and inflammation [65], which can directly damage renal cells, and increase ACR levels [66,67]. This finding aligns with a growing body of evidence suggesting that PAH exposure, including pyrene metabolites like 1-hydroxypyrene, may contribute to renal dysfunction through oxidative stress, inflammation, and tubular injury pathways [20,68]. In animal studies, mice fed pyrene exhibited significant changes in kidney function, indicating renal toxicity [21].

Workers’ wristbands exhibited higher detection frequencies for pesticides compared to community members, including fipronil and pendimethalin, which were only detected in workers’ wristbands, suggesting occupational exposure as a primary source. Both have been associated with renal injury in animal studies [69] and renal disease in human studies among agricultural workers [70,71].

Fipronil is a broad-spectrum insecticide and termiticide that is commonly used in sugarcane plantations [69]. Notably, pendimethalin levels were moderately correlated with higher ACR among workers, suggesting potential nephrotoxic effects through oxidative stress, inflammation, and direct cellular damage [72]. These findings are consistent with literature from Sri Lanka and other CKD-endemic regions implicating pesticide exposure in kidney damage.

Phthalates and consumer-product-related chemicals were ubiquitous. Although community members had higher detection frequencies of some phthalates (i.e., butyl benzyl phthalate and di-n-butyl phthalate), workers exhibited higher concentrations of butyl benzyl phthalate, which showed a weak negative correlation with eGFR-creatinine-cystatin C. The combination of chemical exposures with occupational exposures, such as heat stress, dehydration, and physical exertion [27], could exacerbate the renal effects of chemical exposures like butyl benzyl phthalate [73].

Chlorpyrifos was infrequently detected in our sample, contrasting with prior studies reporting much higher rates, up to 98% among office workers [74] and 91% among farming and non-farming communities in Peru [75]. This discrepancy may reflect seasonal or regional differences in pesticide use. Notably, several pesticides commonly detected in other agricultural studies, such as *p*,*p*′-dichlorodiphenyltrichloroethane (DDT), diazinon, captan, and atrazine, were absent from this study’s wristbands [75,76,77]. This is particularly relevant given that these and similar pesticides have been implicated in CKDu in Sri Lanka [5], Nicaragua [11], and El Salvador [9], where occupational pesticide exposure has been associated with reduced kidney function. Wristbands may underrepresent exposure to low-volatility pesticides (such as organophosphates, pyrethroids, and glyphosate) that are less likely to partition into air or onto the wristband surface, potentially underestimating exposures to some commonly used chemicals. These findings highlight the need for CKDu research to account for evolving agrochemical use patterns and timing of exposure assessment to accurately identify nephrotoxic risk factors.

The detection of flame retardants was low overall. Similar low flame-retardant detection rates have been observed in Senegal and South Africa, possibly due to differences in flammability standards, housing materials, or furniture composition [78]. All participants were exposed to multiple fragrance chemicals, some with potential reproductive or developmental toxicity including benzyl salicylate [79,80,81].

Importantly, observed correlations between chemical exposures and kidney function markers should be interpreted cautiously, as the study was not designed to adjust for potential confounders. The exploratory nature of these findings underscores the need for future studies with larger samples and multivariable analyses to clarify these relationships.

## 5. Limitations

Despite the small sample size and non-random recruitment, this study provides novel insights into occupational versus household exposure pathways. Our sample of 37 women and 45 wristbands may not reflect exposures among women in other regions, though we captured a diverse range of chemicals across fieldwork and household settings. Silicone wristbands assess chemical exposures but do not capture ingestion pathways or distinguish between dermal, inhalation, and ingestion routes. In addition, blank (unworn) wristbands were not included to assess background contamination. Although wristbands were only handled by participants and stored in sealed sterile bags to minimize contamination, the absence of field blanks may reduce confidence in the specificity of detected chemicals. Additional limitations include reliance on self-reported adherence to wearing wristbands, possible seasonal variation since some wristbands were worn in February rather than December and restricting analyses to chemicals with ≥50% detection to reduce spurious associations. Taken together, these limitations indicate our findings should be interpreted as hypothesis-generating, offering important directions for future longitudinal research, including studies of exposure mixtures and assessments outside the sugarcane harvest season to better distinguish occupational from household exposures.

## 6. Conclusions

This study provides novel evidence of widespread environmental chemical exposures among women in agricultural communities in Guatemala, with early signals of association with kidney dysfunction, particularly among field workers, of whom many are already experiencing kidney function decline. The presence of nephrotoxic chemicals like pendimethalin and pyrene, and their correlations with kidney biomarkers, underscores the urgent need to investigate environmental contributions to CKDu among women. The data presented here provide further evidence in support of the hypothesis that the epidemic of CKDu is likely driven by both occupational and community exposures, and it highlights that women are also at risk. This study lays the groundwork for targeted epidemiologic studies and policy interventions to reduce environmental exposures and prevent kidney disease in similar at-risk communities.

## Figures and Tables

**Table 1:** Characteristics and activities among female workers and community members.

Characteristics	Workers (*n* = 19, 51%)	Community Member (*n* = 18, 49%)
Age, years, mean (SD)	38 (8.4)	31 (9.9)
Current work		
- Sugarcane seeder	19 (100%)	N/A
- Does not currently work	N/A	17 (94%)
- Sells dairy products	0 (0%)	1 (6%)
Department of residence		
- Escuintla	18 (100%)	8 (44%)
- Suchitepéquez	0 (0%)	10 (56%)
Education level		
- No school/Primary not complete	7 (39%)	6 (33%)
- Primary complete	3 (17%)	7 (39%)
- More than primary	8 (44%)	5 (28%)
Race		
- Maya/Indigena	2 (11%)	2 (11%)
- Ladino	11 (58%)	16 (89%)
- Not sure	6 (31%)	0 (0%)
Smoking status		
- Never	14 (78%)	13 (75%)
- Past	3 (17%)	0 (0%)
- Current (daily or less than daily)	1 (6%)	1 (6%)
- Refused	0 (0%)	3 (18%)
Primary cook in household	12 (67%)	16 (94%)
Type of fuel used to cook		
- Wood	8 (44%)	8 (47%)
- Gas/Liquid fuel	10 (56%)	9 (53%)
Stove location in compound		
- Inside the main house	8 (44%)	6 (35%) *
- Separate enclosed room	3 (17%)	10 (59%)
- Outside	7 (39%)	1 (6%)
Activities during wristband wearing (7 days)		
Tobacco product use	1 (5%)	0 (0%)
Household member used tobacco product	1 (5%)	1 (6%)
NSAID use	0 (0%)	4 (22%)
Lotion/Body oil use		
- Never	1 (8%)	4 (24%)
- One time	15 (79%)	9 (53%)
- A couple times	3 (16%)	4 (23%)
Nail polish use		
- Never	15 (79%)	17 (100%)
- A couple times	4 (21%)	0 (0%)

* Non-steroidal anti-inflammatory drugs (NSAID) reported included ibuprofen, aspirin, diclofenac, naproxen, and local brands that were identified as an NSAID.

**Table 2:** Detection frequency and number of chemical differences between worker and community member (CM) wristbands by MyExposome chemical classifications.

	Detection Frequency, *n* (%)			Number of Chemicals, Mean (SD), Range		
Chemical Groups ^A^	Worker Wristbands	CM Wristbands	*p*-Value ^	Worker Wristbands	CM Wristbands	*p*-Value ^
Pesticides	25 (93%)	18 (100%)	-	3.08 (1.12), 1–5	2.11 (1.08), 1–5	0.007
PAHs	27 (100%)	18 (100%)	-	6.48 (4.44), 2–17	10.44 (3.54), 3–17	0.002
VOCs	14 (52%)	11 (61%)	0.54	1.29 (0.61), 1–3	1.27 (0.65), 1–3	0.91
Pharmacological	6 (22%)	0 (0%)	-	1 (0), 1–1	-	-
Flame retardants	27 (100%)	17 (94%)	-	1.26 (0.53), 1–3	1.24 (0.44), 1–2	0.98
Chemicals in commerce	27 (100%)	18 (100%)	-	10.90 (1.86), 8–16	11.33 (2.14), 8–17	0.71
Consumer products	27 (100%)	15 (83%)	-	2.14 (0.99), 1–5	1.93 (1.22), 1–5	0.28
Personal care products	27 (100%)	18 (100%)	-	9.74 (2.53), 6–15	8.44 (1.42), 5–11	0.09

PAH, Polycyclic Aromatic Hydrocarbons. VOC, Volatile Organic Compounds. ^A^ Chemicals may be classified in more than 1 category, see Supplemental Table S1 for classifications. ^ Differences between participant groups were tested with linear mixed-effects regression models to account for the dependency between repeated measurements on the same participant. Differences were not tested with high detection frequencies in both groups, denoted as “-”.

**Table 3:** Descriptive statistics of chemical concentrations measured in wristbands with ≥ 30% detected in either participant group (Subset 1 Chemicals) and detection and concentration (ng/g silicone) differences between participant groups.

	Worker Wristbands (*n* = 27)		Community Member Wristbands (*n* = 18)				MyExposome Classification ^A^				
Chemical	% Detected	Median (IQR)	% Detected	Median (IQR)	Pesticide	PAH	VOC	Flame Retardant	Chemical in Commerce	Consumer Product	Personal Care Product
Benzyl salicylate	100	2550 (1310, 4440)	100	3290 (930, 9340)							X
Diisobutyl phthalate	100	1930 (995, 4110)	100	1465 (785, 3000)					X		
Galaxolide	100	6330 (4110, 8230)	100	8775 (4670, 11,500)					X		X
Lilial	100	668 (323, 1210)	100	910.5 (318, 1420)							X
Pyrene	100	68.7 (41.1, 106)	100	116.55 (57.3, 264) ^		X			X		
Tonalide	100	283 (141, 668)	100	280.5 (112, 467)							X
Triphenyl phosphate	100	144 (113, 249)	94	297 (102, 4260) ^				X	X		
Diethyl phthalate	96	774 (213, 2180)	94	465 (182, 658)					X		
Bis(2-ethylhexyl) phthalate	93	422 (283, 985)	100	503 (233, 1050)					X		
Anthracene	85	56.6 (30.4, 99)	83	62.0 (42.6, 116)		X					
Caffeine	85	1360 (739, 2130)	44 ^	352 (197.5, 406.5) ^						X	
Ethylene brassylate	81	1090 (404, 2370)	78	1260 (340, 4240)							X
Di-n-butyl phthalate	78	3090 (2350, 6680)	100 *	5870 (2350, 10,900)					X		X
Di-n-nonyl phthalate	74	490.5 (329, 1018)	61	966 (504, 1550)					X		
Diuron metabolite ^B^	63	219 (126, 283)	0	0 (0, 0)					X		
Pendimethalin	63	463 (226, 591)	0	0 (0, 0)	X						
Benzophenone	56	30.9 (21.3, 59.1)	33	90.75 (10.2, 205)					X		X
β-Ionone	56	147 (108, 249)	83 *	202 (65.8, 548)							X
Amyl cinnamal	48	206 (118, 334)	28	76.4 (62, 110)							X
Benzyl benzoate	44	1235 (629.5, 5805)	39	773 (140, 9690)	X						
Butyl benzyl phthalate	44	116 (62.65, 261)	72 *	91.2 (72.2, 158)					X		
Naphthalene	41	5.66 (4.63, 6.43)	11 ^	13.37 (4.24, 22.5)		X	X				
2-Methylphenanthrene	41	16.0 (12.9, 28.3)	61	31.8 (20.5, 50.4)		X					
Benz[a]anthracene	37	21.0 (14.9, 36.0)	94 ^	36.1 (18.4, 47.7)		X					
Musk ketone	37	360 (334, 488)	11 *	834.5 (279, 1390)							X
Fluorene	33	16.0 (11.1, 16.5)	72 ^	16.7 (10.9, 27.6)		X					
Cyclopenta[cd]pyrene	33	22.5 (16.5, 30.9)	56	44.85 (38.2, 65.9) ^		X					
Permethrin	33	77.1 (74.6, 309)	33	234.5 (59.4, 580)	X						
Citral A	33	46.3 (38.4, 64.3)	6 ^	22.7 (22.7, 22.7)						X	X
1-Methylphenanthrene	30	16.8 (9, 22.9)	44	19.6 (17.2, 28.45)		X					
B-citronellol	30	98.5 (58.65, 247)	22	43.5 (30.75, 98.6)						X	X
Fipronil	30	114 (83.55, 136)	0	0 (0, 0)	X						
Benzo[a]pyrene	26	6.94 (5.14, 15.7)	61 ^	21.2 (10.8, 28.6)		X					
Fluoranthene	26	38.6 (17.5, 141)	61 ^	119 (67.7, 322) *		X					
Biphenyl	26	9.67 (8.44, 15.8)	67 ^	8.09 (5.09, 10.36)	X				X		
Chrysene	22	38.65 (25.3, 64.3)	50 *	50.4 (24.8, 89.1)		X					
9-Fluorenone	22	36.75 (21.1, 54.5)	39	29.8 (15.5, 58.1)		X			X		
1-Methylnaphthalene	19	6.3 (5.3, 6.6)	56 ^	3.8 (3.2, 4.9)		X	X		X		
Benzo[a]fluorene	19	22.6 (11.7, 25.5)	44 *	26.95 (22, 35.3)		X					
Phenanthrene	19	72 (51.4, 74.6)	44 *	211 (76.85, 325.5) ^		X					
Benzo[ghi]perylene	11	8.48 (3.34, 70.4)	33 *	12.74 (8.06, 27.4)		X					

^A^ “X” denotes that the detected chemical is classified in the MyExposome category. No Pharmacologic chemicals had ≥30% detected in either group thus this category was removed from the table. ^B^ 3,4-Dichlorophenyl isocyanate. ^ *p*-values ≤ 0.05. * *p*-values between 0.06–0.10.

**Table 4:** Clinical data and markers of kidney function among workers and community members.

	Workers (*n* = 19, 51%)	Community Members (*n* = 18, 49%)
Diastolic blood pressure, mmHg	72 (70, 80)	74 (69, 77)
Systolic blood pressure, mmHg	110 (110, 120)	109 (104, 117)
Hypertension ^A^, *n* (%)	0	2 (11%)
Body mass index, kg/m^2^	26.9 (23.4, 29.2)	26.4 (22.7, 32.1)
HbA1c, %	4.7 (4.4, 4.9)	5.2 (5.1, 5.5) *
Hematocrit, %	36.0 (34, 39)	39.5 (38, 41) *
Hemoglobin, g/dL	12.2 (11.6, 13.3)	13.6 (13.3, 14.0) *
eGFR-creatinine ^B^	114.88 (93.70, 119.99)	125.34 (106.77, 130.94) *
- 60–<90, *n* (%)	3 (17%)	1 (6%)
- <60, *n* (%)	1 (6%)	0
eGFR-creatinine-cystatin C ^B^	91.97 (78.66, 105.91)	117.27 (105.22, 125.95) *
- 60–<90, *n* (%)	8 (44%)	0
- <60, *n* (%)	1 (6%)	0
Urine ACR, mg/g	16.2 (8.6, 29.2)	12.3 (6.6, 18.7)
- ≥30, *n* (%)	4 (21%)	2 (11%)

Median (25th, 75th percentiles) are presented unless otherwise stated. eGFR: estimated glomerular filtration rate. ACR: Albumin to Creatinine Ratio. ^A^ Stage 1 hypertension is defined as having systolic blood pressure from 130–139 or having diastolic blood pressure from 80–89. ^B^ ml/min/1.73 m^2^. * Denotes significance at *p* < 0.05.

**Table 5:** Spearman correlation coefficients between markers of kidney function and chemical concentrations detected in ≥ 50% of wristbands stratified by participant group.

	eGFR-Creatinine	eGFR-Creatinine-Cystatin C	ACR
Diethyl phthalate			Community Member
			r = 0.44, *p* = 0.09
Butyl benzyl phthalate		Worker	
		r = −0.57, *p* = 0.05	
Pyrene			Worker
			r = 0.57, *p* = 0.003
β-ionone	Worker	Worker	Worker
	r = −0.40, *p* = 0.14	r = −0.36, *p* = 0.18	r = 0.43, *p* = 0.14
Benzyl salicylate			Worker
			r = 0.34, *p* = 0.10
Pendimethalin			Worker
			r = 0.55, *p* = 0.03

eGFR: estimated glomerular filtration rate. ACR: Albumin to Creatinine Ratio. Correlation coefficients and *p*-values presented for *p*-values < 0.20.

## Data Availability

The original data presented in the study are openly available in OSFHome, OSF|OSF.

## Supplementary Materials

The following supporting information can be downloaded at: https://www.mdpi.com/article/10.3390/toxics13090761/s1, Table S1: Descriptive statistics of chemical concentrations (ng/g silicone) measured in silicone wristbands among workers and community members; Table S2: Chemicals detected in at least 50% of the wristbands (Subset 2 Chemicals); Figure S1: Log-transformed concentration ranges of polycyclic aromatic hydrocarbon (PAH) chemical concentrations displayed using a box plot with the lower 5th and upper 95th percentile represented by the whiskers, the 25th and 75th percentiles represented by the box edges, and the median represented by a line bisecting each box; Figure S2: Log-transformed concentration ranges of personal care products, chemicals in commerce, triphenyl phosphate (TPP), and caffeine chemical concentrations displayed using a box plot with the lower 5th and upper 95th percentile represented by the whiskers, the 25th and 75th percentiles represented by the box edges, and the median represented by a line bisecting each box; Figure S3: Log-transformed concentration ranges of pesticides and phthalate chemical concentrations displayed using a box plot with the lower 5th and upper 95th percentile represented by the whiskers, the 25th and 75th percentiles represented by the box edges, and the median represented by a line bisecting each box.

## Abbreviations

The following abbreviations are used in this manuscript:

CKDu	Chronic kidney disease of unknown origin
PAH	Polycyclic aromatic hydrocarbons
eGFR	Estimated glomerular filtration rate
ACR	Albumin to Creatinine ratio
BMI	Body mass index
PTFE	Polytetrafluoroethylene
GC-MS	Gas chromatography–mass spectrometry
VOC	Volatile organic chemicals
TPP	Triphenyl phosphate
