# Supplementary material for: Measuring Environmental Chemical Burden with Wristbands: Implications for Kidney Health Among Women in Rural Guatemala
Source: Toxics. 2025 Sep 8;13(9):761. doi: 10.3390/toxics13090761 (PMC12474246; doi:10.3390/toxics13090761)
Supplement: Supplementary file 1 [file toxics-13-00761-s001.zip › toxics-3838123-supplementary.pdf]

Supplementary Material

Measuring Environmental Chemical Burden with Wristbands: Implications for Kidney Health Among Women in Rural Guatemala

Jaime Butler-Dawson [1,2*], Grant Erlandson [3], Diana Jaramillo [1,2], Karely Villarreal Hernandez [1], Laura Calvimontes [3], Lyndsay Krisher [1,2], Miranda Dally [1,2], Stephen Brindley [1,2], Daniel Pilloni [4], Alex Cruz [4], Alison K. Bauer [1], Richard J. Johnson [5], Lee S. Newman [1,2,6,7], Joshua Schaeffer [3], John L. Adgate [1,2], Kim A. Anderson [8] and Katherine A. James [1,2]

1. Department of Environmental and Occupational Health, Colorado School of Public Health, University of Colorado Anschutz Campus, Aurora, CO, USA 80045
2. Centers for Health Work and Environment, Colorado School of Public Health, University of Colorado, Anschutz Campus, Aurora, CO, USA 80045
3. Department of Environmental and Radiological Health Sciences, Colorado State University, Fort Collins, CO, USA 80523
4. Grupo Pantaleon, Guatemala City, Guatemala
5. Division of Renal Diseases and Hypertension, University of Colorado School of Medicine, Anschutz Medical Campus, Aurora, CO, USA 80045
6. Department of Epidemiology, Colorado School of Public Health, University of Colorado, Anschutz Campus, Aurora, CO, USA 80045.
7. Division of Pulmonary Sciences and Critical Care Medicine, Department of Medicine, School of Medicine, University of Colorado, Anschutz Campus, Aurora, CO, USA 80045.
8. Department of Environmental and Molecular Toxicology, Oregon Status University, Corvallis, OR, USA 97331

* Correspondence: jaime.butler-dawson@cuanschutz.edu

Supplemental Table S1: *Descriptive statistics of chemical concentrations (ng/g) measured in silicone wristbands among workers and community members.*

		**Worker Wristbands (n = 27)**			**Community Member Wristbands (n = 18)**			**MyExposome Classification**							
**Chemical**	CASN	% Detected	Median (IQR)	(5th, 95th)	% Detected	Median (IQR)	(5th, 95th)	Pesticide	PAH	VOC	Pharma-cologic	Flame Retardant	Chemicals in Commerce	Consumer Product	Personal Care Product
Benzyl salicylate	118-58-1	100	2550 (1310, 4440)	(116, 7460)	100	3290 (930, 9340)	(403, 42600)								X
Diisobutyl phthalate	84-69-5	100	1930 (995, 4110)	(514, 9770)	100	1465 (785, 3000)	(109, 10600)						X		
Galaxolide	1222-05-5	100	6330 (4110, 8230)	(2110, 14900)	100	8775 (4670, 11500)	(1470, 36100)						X		X
Lilial	80-54-6	100	668 (323, 1210)	(175, 3480)	100	910.5 (318, 1420)	(80.5, 2760)								X
Pyrene	129-00-0	100	68.7 (41.1, 106)	(21.5, 208)	100	116.55 (57.3, 264)	(38.2, 612)		X				X		
Tonalide	1506-02-1	100	283 (141, 668)	(44.6, 2340)	100	280.5 (112, 467)	(30.6, 1220)								X
Triphenyl phosphate	115-86-6	100	144 (113, 249)	(79.7, 505)	94	297 (102, 4260)	(42.4, 19300)					X	X		
Diethyl phthalate	84-66-2	96	774 (213, 2180)	(52.8, 3870)	94	465 (182, 658)	(146, 6790)						X		
Bis(2-ethylhexyl) phthalate	117-81-7	93	422 (283, 985)	(116, 2260)	100	503 (233, 1050)	(128, 5730)						X		
Benz[a]anthracene	56-55-3	37	21 (14.9, 36)	(12.3, 120)	94	36.1 (18.4, 47.7)	(13.2, 129)		X						
Caffeine	58-08-2	85	1360 (739, 2130)	(283, 5630)	44	352 (197.5, 406.5)	(159, 501)							X	
Ethylene brassylate	105-95-3	81	1090 (404, 2370)	(238, 6330)	78	1260 (340, 4240)	(177, 17400)								X
Di-n-butyl phthalate	84-74-2	78	3090 (2350, 6680)	(1690, 11600)	100	5870 (2350, 10900)	(620, 28600)						X		X
Di-n-nonyl phthalate	84-76-4	74	490.5 (329, 1018)	(236.5, 6045)	61	966 (504, 1550)	(255, 14800)						X		
Diuron Metabolite	102-36-3	63	219 (126, 283)	(25.5, 900)	0	0 (0,0)	(0,0)						X		
Pendimethalin	40487-42-1	63	463 (226, 591)	(95.1, 2630)	0	0 (0,0)	(0,0)	X							
Benzophenone	119-61-9	56	30.9 (21.3, 59.1)	(14.1, 226)	33	90.75 (10.2, 205)	(6.44, 615)						X		X
β-Ionone	79-77-6	56	147 (108, 249)	(42.2, 1580)	83	202 (65.8, 548)	(40.3, 891)								X
Amyl cinnamal	122-40-7	48	206 (118, 334)	(35.2, 1260)	28	76.4 (62, 110)	(45.1, 144)								X
Benzyl benzoate	120-51-4	44	1235 (630, 5805)	(103, 8800)	39	773 (140, 9690)	(97.6, 19400)	X							

Butyl benzyl phthalate	85-68-7	44	116 (62.65, 261)	(16.9, 977)	72	91.2 (72.2, 158)	(50.9, 1170)				X		
Anthracene	120-12-7	85	56.6 (30.4, 99)	(13.4, 216)	83	62 (42.6, 116)	(36.1, 318)		X				
Fluorene	86-73-7	33	16 (11.1, 16.5)	(6.17, 27.9)	72	16.7 (10.9, 27.6)	(4.06, 45.1)		X				
2-Methylphenanthrene	2531-84-2	41	16 (12.9, 28.3)	(9.77, 91.5)	61	31.8 (20.5, 50.4)	(14.2, 93.4)		X				
Musk Ketone	81-14-1	37	360 (334, 488)	(88, 4110)	11	834.5 (279, 1390)	(279, 1390)						X
Citral A	5392-40-5	33	46.3 (38.4, 64.3)	(30.9, 103)	6	22.7 (22.7, 22.7)	(22.7, 22.7)					X	X
Benzo[a]pyrene	50-32-8	26	6.94 (5.14, 15.7)	(4.04, 95)	61	21.2 (10.8, 28.6)	(5.73, 48.3)		X				
Fluoranthene	206-44-0	26	38.6 (17.5, 141)	(14.9, 151)	61	119 (67.7, 322)	(19.1, 677)		X				
Permethrin	52645-53-1	33	77.1 (74.6, 309)	(40.4, 566)	33	234.5 (59.4, 580)	(27.6, 637)	X					
Cyclopenta[cd]pyrene	27208-37-3	33	22.5 (16.5, 30.9)	(7.71, 51.4)	56	44.85 (38.2, 65.9)	(11.9, 197)		X				
B-citronellol	106-22-9	30	98.5 (58.7, 247)	(46.3, 309)	22	43.5 (30.8, 98.6)	(29.7, 142)					X	X
Fipronil	120068-37-3	30	114 (83.55, 136)	(69.4, 208)	0	0 (0,0)	(0,0)	X					
2,4-Di-tert-butylphenol	96-76-4	26	41.1 (20.6, 48.5)	(15.4, 51.4)	6	2.12 (2.12, 2.12)	(2.12, 2.12)				X		
1-Methylnaphthalene	90-12-0	19	6.33 (5.28, 6.63)	(3.03, 6.83)	56	3.82 (3.18, 4.88)	(2.33, 12.4)		X	X	X		
Biphenyl	92-52-4	26	9.67 (8.44, 15.8)	(4.93, 28.3)	67	8.09 (5.09, 10.36)	(3.82, 24.4)	X			X		
Chrysene	218-01-9	22	38.65 (25.3, 64.3)	(16.7, 183)	50	50.4 (24.8, 89.1)	(17, 116)		X				
Linalool	78-70-6	26	116 (64.3, 134)	(45.7, 341)	0	0 (0,0)	(0,0)	X					X
Triclosan	3380-34-5	26	100 (23.1, 306)	(18.8, 3480)	6	14.7 (14.7, 14.7)	(14.7, 14.7)				X		X
1-Methylphenanthrene	832-69-9	30	16.8 (9, 22.9)	(8.23, 30.6)	44	19.6 (17.2, 28.45)	(8.38, 54.8)		X				
Benzo[a]fluorene	238-84-6	19	22.6 (11.7, 25.5)	(11.3, 45.7)	44	26.95 (22, 35.3)	(5.09, 80.5)		X				
Coumarin	91-64-5	22	128.75 (69.4, 165)	(59.1, 229)	22	83.05 (52, 118.5)	(50.9, 124)					X	X
Phenanthrene	85-01-8	19	72 (51.4, 74.6)	(51.4, 170)	44	211 (76.85, 325.5)	(74.1, 644)		X				
A-Ionone	127-41-3	19	211 (193, 257)	(149, 437)	6	104 (104, 104)	(104, 104)						X
9-Fluorenone	486-25-9	22	36.75 (21.1, 54.5)	(13.6, 56.6)	39	29.8 (15.5, 58.1)	(11.2, 119)		X		X		

Cashmeran	33704-61-9	19	197 (139, 246)	(64.3, 950)	28	38.2 (34, 47.7)	(20, 78.5)								X
Benzo[ghi]perylene	191-24-2	11	8.48 (3.34, 70.4)	(3.34, 70.4)	33	12.74 (8.06, 27.4)	(5.25, 30.2)		X						
Benzo[b]fluorene	243-17-4	15	17.6 (12.5, 24)	(12.3, 25.5)	28	31.4 (11, 31.8)	(7.43, 32.2)		X						
1-Methylpyrene	2381-21-7	15	13.74 (7.455, 71)	(6.43, 123)	22	27.2 (22.7, 33.3)	(18.9, 38.7)		X						
Butylated hydroxytoluene	128-37-0	15	100.3 (56.5, 564.5)	(56.3, 985)	17	67.9 (21.2, 2830)	(21.2, 2830)						X	X	
Acenaphthylene	208-96-8	11	33.4 (33.2, 41.6)	(33.2, 41.6)	22	33.7 (16.36, 59.7)	(7.22, 77.5)		X						
Benzo[k]fluoranthene	207-08-9	0	0 (0,0)	(0,0)	22	38.4 (29.7, 49.4)	(23.3, 58.1)		X						
Clomazone	81777-89-1	11	46.3 (33.4, 131)	(33.4, 131)	0	0 (0,0)	(0,0)	X							
Cypermethrin-2	52315-07-8	11	252 (221, 283)	(221, 283)	0	0 (0,0)	(0,0)	X							
Eugenol	97-53-0	11	31 (28.1, 151)	(28.1, 151)	0	0 (0,0)	(0,0)				X		X	X	X
N,N-Diethyl-m-toluamide	134-62-3	11	49.8 (35.2, 83.2)	(35.2, 83.2)	28	145 (105, 148)	(59.4, 286)	X							
PBDE 49	243982-82-3	11	111 (38.6, 134)	(38.6, 134)	0	0 (0,0)	(0,0)					X			
Naphthalene	91-20-3	41	5.66 (4.63, 6.43)	(3.64, 15.8)	11	13.37 (4.24, 22.5)	(4.24, 22.5)		X	X					
1,6-Dimethylnaphthalene	575-43-9	7	2.6705 (0.77, 4.57)	(0.771, 4.57)	11	5.31 (2.97, 7.64)	(2.97, 7.64)		X	X					
2,6-Dimethoxyphenol	91-10-1	7	61.7 (58, 65.4)	(58, 65.4)	17	64.4 (62, 116)	(62, 116)						X	X	
2,6-Dimethylnaphthalene	581-42-0	0	0 (0,0)	(0,0)	11	15.08 (4.65, 25.5)	(4.65, 25.5)		X						
Butylated hydroxyanisole	25013-16-5	7	1171.95 (23.9, 2320)	(23.9, 2320)	0	0 (0,0)	(0,0)				X				X
Chlorpyrifos	2921-88-2	7	2085 (1340, 2830)	(1340, 2830)	17	129 (38.2, 169)	(38.2, 169)	X							
Di-n-octyl phthalate	117-84-0	7	14100 (13900, 14300)	(13900, 14300)	11	819.5 (349, 1290)	(349, 1290)						X		
Permethrin II	999046-03-6	7	153.75 (54.5, 253)	(54.5, 253)	11	248.45 (50.9, 446)	(50.9, 446)	X							
TCPP	26248-87-3	7	62.25 (52.5, 72)	(52.5, 72)	0	0 (0,0)	(0,0)					X	X		
4H-cyclopenta [def]phenanthren-4-one	5737-13-3	0	0 (0,0)	(0,0)	11	317.5 (216, 419)	(216, 419)		X				X		
Benzothiazole	95-16-9	4	130 (130, 130)	(130, 130)	0	0 (0,0)	(0,0)						X		

Buprofezin	69327-76-0	4	92.6 (92.6, 92.6)	(92.6, 92.6)	0	0 (0,0)	(0,0)	X							
Celestolide	13171-00-1	4	38.6 (38.6, 38.6)	(38.6, 38.6)	6	77.3 (77.3, 77.3)	(77.3, 77.3)							X	X
Cyclopentadecanone	502-72-7	4	155 (155, 155)	(155, 155)	0	0 (0,0)	(0,0)								X
Drometrizole	2440-22-4	4	283 (283, 283)	(283, 283)	0	0 (0,0)	(0,0)						X	X	
Exaltolide [15-Pentadecanolide]	106-02-5	4	1000 (1000, 1000)	(1000, 1000)	6	3820 (3820, 3820)	(3820, 3820)								X
Geraniol	106-24-1	4	162 (162, 162)	(162, 162)	0	0 (0,0)	(0,0)							X	X
Hexazinone	51235-04-2	4	242 (242, 242)	(242, 242)	0	0 (0,0)	(0,0)	X							
Hydroxy-citronellal	107-75-5	4	387 (387, 387)	(387, 387)	0	0 (0,0)	(0,0)								X
Lyral	31906-04-4	4	123 (123, 123)	(123, 123)	0	0 (0,0)	(0,0)								X
Methyleugenol	93-15-2	4	118 (118, 118)	(118, 118)	0	0 (0,0)	(0,0)								X
PBDE 51	189084-57-9	4	154 (154, 154)	(154, 154)	0	0 (0,0)	(0,0)					X			
Piperonyl butoxide	51-03-6	4	334 (334, 334)	(334, 334)	0	0 (0,0)	(0,0)	X							
Quinoline	91-22-5	4	24.2 (24.2, 24.2)	(24.2, 24.2)	0	0 (0,0)	(0,0)						X		
Thymol	89-83-8	4	25.7 (25.7, 25.7)	(25.7, 25.7)	0	0 (0,0)	(0,0)				X				
Transfluthrin	118712-89-3	4	201 (201, 201)	(201, 201)	0	0 (0,0)	(0,0)	X							
Tributyl phosphate	126-73-8	4	33.4 (33.4, 33.4)	(33.4, 33.4)	0	0 (0,0)	(0,0)					X	X		
Retene	483-65-8	15	25.34 (4.50, 66.85)	(0.514, 91.5)	6	18.6 (18.6, 18.6)	(18.6, 18.6)		X						
2,6-Diethylnaphthalene	59919-41-4	0	0 (0,0)	(0,0)	6	4.24 (4.24, 4.24)	(4.24, 4.24)		X						
2-Methylnaphthalene	91-57-6	7	2.625 (2.42, 2.83)	(2.42, 2.83)	0	0 (0,0)	(0,0)		X	X			X		
2,4-Bis(alpha,alpha-dimethylbenzyl)phenol	2772-45-4	0	0 (0,0)	(0,0)	6	78.5 (78.5, 78.5)	(78.5, 78.5)						X		
3,6-Dimethylphenanthrene	1576-67-6	4	66.8 (66.8, 66.8)	(66.8, 66.8)	0	0 (0,0)	(0,0)		X						
6-Methylchrysene	1705-85-7	4	387 (387, 387)	(387, 387)	0	0 (0,0)	(0,0)		X						
2-Methoxy-4-methylphenol	93-51-6	0	0 (0,0)	(0,0)	6	93 (93, 93)	(93, 93)							X	

4-Chloro-3,5-dimethylphenol	88-04-0	0	0 (0,0)	(0,0)	6	221 (221, 221)	(221, 221)				X		
7,12-Dimethylbenz [a]anthracene	57-97-6	4	264 (264, 264)	(264, 264)	0	0 (0,0)	(0,0)		X				
4-Tert-butylphenol	98-54-4	0	0 (0,0)	(0,0)	6	38.7 (38.7, 38.7)	(38.7, 38.7)				X	X	
Triphenylene	217-59-4	4	1.62 (1.62, 1.62)	(1.62, 1.62)	0	0 (0,0)	(0,0)		X				
Benzyl cinnamate	103-41-3	0	0 (0,0)	(0,0)	11	131.65 (85.3, 178)	(85.3, 178)					X	X
Dicyclohexyl phthalate	84-61-7	0	0 (0,0)	(0,0)	6	310 (310, 310)	(310, 310)				X		
Diphenylamine	122-39-4	0	0 (0,0)	(0,0)	6	14.6 (14.6, 14.6)	(14.6, 14.6)	X					
Ethofenprox	80844-07-1	0	0 (0,0)	(0,0)	6	142 (142, 142)	(142, 142)	X					
Fluazifop-p-butyl	79241-46-6	0	0 (0,0)	(0,0)	6	78.8 (78.8, 78.8)	(78.8, 78.8)	X					
Tricresylphosphate, meta-	563-04-2	0	0 (0,0)	(0,0)	11	177.2 (64.4, 290)	(64.4, 290)			X	X		
Tricresylphosphate, ortho-	78-30-8	0	0 (0,0)	(0,0)	6	93.4 (93.4, 93.4)	(93.4, 93.4)			X	X		
Triethyl phosphate	78-40-0	0	0 (0,0)	(0,0)	6	38.2 (38.2, 38.2)	(38.2, 38.2)			X	X		
Tri-p-tolyl phosphate	78-32-0	0	0 (0,0)	(0,0)	6	32.6 (32.6, 32.6)	(32.6, 32.6)				X		

*3,4-Dichlorophenyl isocyanate.

Supplemental Table S2: *Chemicals detected in at least 50% of the wristbands (Subset 2 Chemicals).*

Chemical Name	Percent Detected, %
Benzyl salicylate	100
Diisobutyl phthalate	100
Galaxolide	100
Lilial	100
Pyrene	100
Tonalide	100
Triphenyl phosphate (TPP)	98
Bis(2-ethylhexyl) phthalate	96
Diethyl phthalate	95
Di-n-butyl phthalate	87
Anthracene	84
Ethylene brassylate	80
Caffeine	69
Di-n-nonyl phthalate	69
β-Ionone	67
Benz[a]anthracene	60
Butyl benzyl phthalate	55

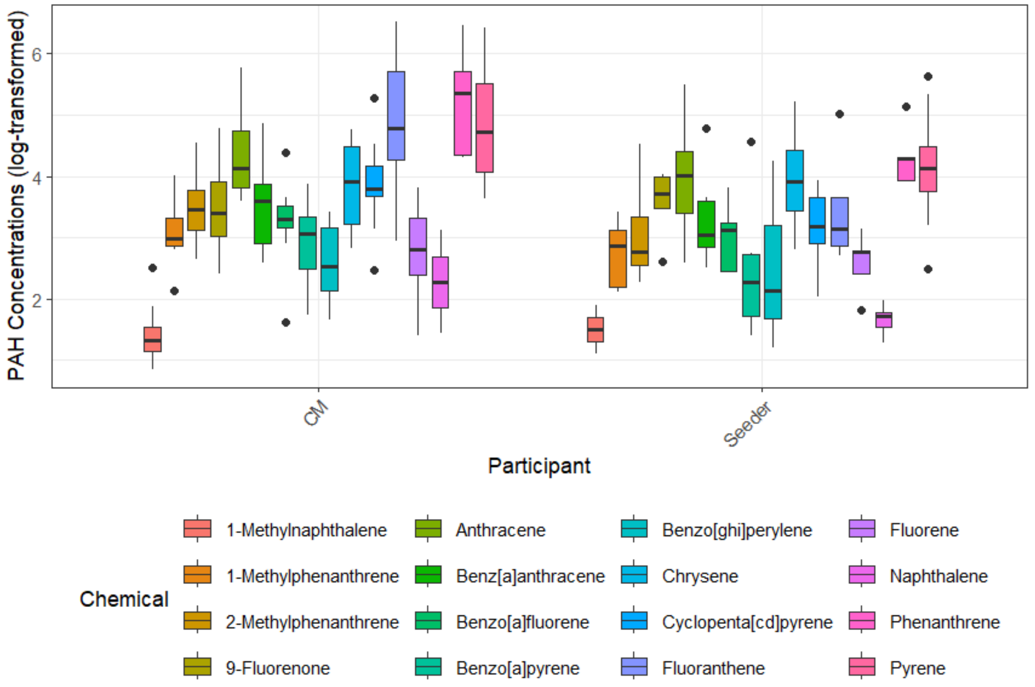

Supplemental Figure S1: *Log-transformed concentration ranges of polycyclic aromatic hydrocarbons (PAH) chemical concentrations displayed using a box plot with the lower 5th and upper 95th percentile represented by the whiskers, the 25th and 75th percentiles represented by the box edges, and the median represented by a line bisecting each box.*

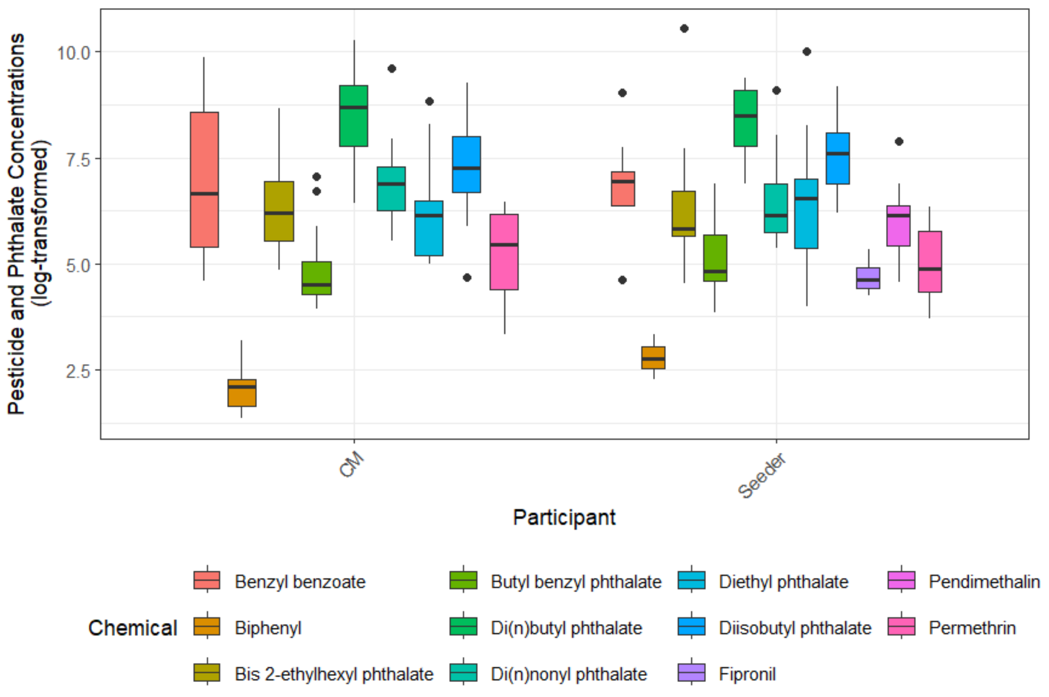

Supplemental Figure S2: *Log-transformed concentration ranges of pesticides and phthalate chemical concentrations displayed using a box plot with the lower 5th and upper 95th percentile represented by the whiskers, the 25th and 75th percentiles represented by the box edges, and the median represented by a line bisecting each box.*

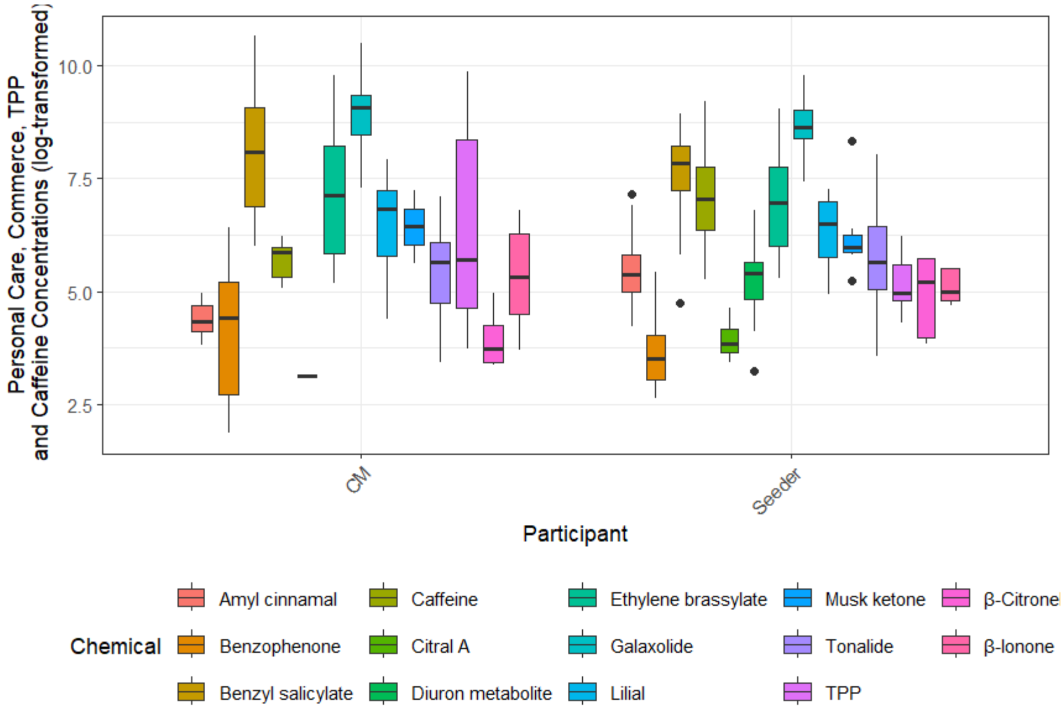

Supplemental Figure S3: *Log-transformed concentration ranges of personal care products, chemicals in commerce, triphenyl phosphate (TPP), and caffeine chemical concentrations displayed using a box plot with the lower 5th and upper 95th percentile represented by the whiskers, the 25th and 75th percentiles represented by the box edges, and the median represented by a line bisecting each box.*
